# Spatial Trajectory Tracking of Wall-Climbing Robot on Cylindrical Tank Surface Using Backstepping Sliding-Mode Control

**DOI:** 10.3390/mi14030548

**Published:** 2023-02-26

**Authors:** Jiameng Xue, Jingyu Chen, Alexsandru Stancu, Xingsong Wang, Jie Li

**Affiliations:** 1 College of Automation, Nanjing University of Posts and Telecommunications, Nanjing 210003, China; 2 School of Engineering, The University of Manchester, Manchester M13 9PL, UK; 3 School of Mechanical Engineering, Southeast University, Nanjing 211189, China

**Keywords:** tank inspection, climbing robot, positioning, trajectory tracking, backstepping control, sliding-mode control

## Abstract

Wall-climbing robots have been well-developed for storage tank inspection. This work presents a backstepping sliding-mode control (BSMC) strategy for the spatial trajectory tracking control of a wall-climbing robot, which is specially designed to inspect inside and outside of cylindrical storage tanks. The inspection robot is designed with four magnetic wheels, which are driven by two DC motors. In order to achieve an accurate spatial position of the robot, a multisensor-data-fusion positioning method is developed. The new control method is proposed with kinematics based on a cylindrical coordinate system as the robot is moving on a cylindrical surface. The main purpose is to promote a smooth and stable tracking performance during inspection tasks, under the consideration of the robot’s kinematic constraints and the magnetic restrictions of the adhesion system. The simulation results indicate that the proposed sliding mode controller can quickly correct the errors and global asymptotic stability is achieved. The prototype experimental results further validate the advancement of the proposed method; the wall-climbing robot can track both longitudinal and horizontal spatial trajectories stably with high precision.

## 1. Introduction

As kinds of pressure vessels, cylindrical storage tanks are commonly used for the storage of oil or radioactive chemical liquids. Generally, storage tanks are welded from pieces of cambered plates. Thus, weld lines are distributed on the surface of the tank. Since the special media inside the vessels could cause corrosion or defects on the welds, such as pits, cracks and holes, it is very necessary to regularly check the security performance of the tanks. Currently, inspection and maintenance work are all carried out manually, usually based on ultrasound or other nondestructive techniques (NDTs). This kind of inspection requires workers to move slowly and precisely over surface, so the whole process takes a long time. With the development of robotic technologies, many kinds of inspection robots have been designed to take the place of manual maintenance [1,2,3,4,5,6]. As the robot can carry devices and instruments for detection or reconstruction, this leads to a reduction in inspection time and costs, as well as an improvement of the accuracy and repeatability.

During the inspection process, the mobile robot is required to follow the weld lines or to track a given trajectory. Thus, the accuracy of trajectory tracking and fast responses are required. In order to achieve this, numerous control strategies have been developed for tracking control of wall-climbing robots. For example, Kuo et al. [7] used a traditional PID controller for trajectory tracking of a wheeled wall climbing robot; Gimenez et al. [8] chose an adaptive control method to reduce the effect of gravity factors. Dian et al. [9] presented an improved dual-heuristic dynamic programming method for magnetic wheeled mobile robots. In [10], the authors introduced a nonlinear model predictive control (NMPC)-based tracking control method for a novel wall-climbing robot.

Sliding-mode control (SMC) is another control method that has been successfully applied in tracking control for wall-climbing robots. The key advantages of SMC are the robustness to system uncertainties and external disturbances, easy implementation and fast response. These features make SMC an attractive control approach for the tracking problem of wall-climbing robots, as the robot system is affected by adsorption force and gravity. Wu et al. [11] designed a sliding-mode controller with integral action for the tracking control of a climbing robot. Xin et al. [12] proposed a backstepping adaptive fuzzy sliding-mode control (BFASMC) method to improve the robustness and control precision of trajectory tracking for a wall-climbing robot. In [13], an adaptive extended state-observer-based nonsingular terminal sliding-mode control (NTSMC) was presented for aircraft-skin inspection robots.

In this paper, a differential wheeled wall-climbing robot is specially designed for the inspection of cylindrical storage tanks, which is equipped with an onboard camera and four magnetic wheels driven by two DC motors. This robot is subjected to nonholonomic constraints. As we know, a proper choice of tracking errors can eliminate the geometric constraints. In order to measure the spatial coordinates of the robot’s position, a spatial positioning method based on multisensor data fusion is proposed. To verify the accuracy and stability of the positioning method, positioning stability experiments are performed, and the results are analysed. Considering that the robot is moving on the surface of a cylinder, a cylindrical coordinate system is chosen to represent the three-dimensional position of the robot as well as the tracking errors. In this case, a hybrid control strategy is proposed for trajectory tracking problems based on the combination of backstepping control and sliding-mode control. The simulation results indicate that the performance of the control system is guaranteed. The prototype experimental results further validate the advancement of the proposed method.

This paper is organized as follows. Section 2 introduces the mechanical design of the climbing robot. Section 3 describes the three-dimensional positioning of the robot, including the multisensor positioning and positioning accuracy analysis. In Section 4, we present the strategy of BSMC for trajectory tacking. In addition, a numerical simulation is included. Section 5 demonstrates the real prototype experiments, and the experimental results are discussed.

## 2. Robot System Design

To ensure the safety and stability of wall-climbing robots, a sufficient adsorption capacity has to be considered first. According to different working environments, various adsorption techniques can be selected, such as negative pressure adsorption [14,15], bionic adsorption [16,17], thrust adsorption [18,19] and magnetic adsorption [20,21,22]. Generally, permanent magnet adsorption is used for metal walls, which can provide a large adsorption force without requiring external energy consumption. Many types of magnetic crawler robots have been investigated, such as climbing robots with magnetic wheels [23,24,25], omnidirectional climbing robots [10,26,27] and magnetic crawler climbing robots [28,29,30,31]. Among them, wall-climbing robots with magnetic wheels are widely used in the inspection task of storage tanks, bridges and ships. These kinds of wall-climbing robots are designed to carry a variety of equipment and instruments instead of manual operations. Some different wall-climbing robots have been investigated for storage tank inspection and maintenance. For example, an autonomous climbing robot was designed in [32], which can work inside and outside spherical storage tanks, a noncontacted permanent-magnet-adsorbed wall-climbing robot was introduced in [33] for the inspection of the spherical tank.

In our earlier studies, a series of wall-climbing robots were developed and tested [34,35], which could effectively move on tank surfaces. In this section, a new design of a wall-climbing robot is proposed, which is equipped with an onboard camera and four magnetic wheels driven by two motors. One main advantage of differential wheeled robots is that they can change the heading direction by adjusting the angular velocities of two driving motors, such as following a curvilinear trajectory or turning around. The modular magnetic wheels provide a reliable adsorption force for the climbing robot, and these wheels can also be easily installed and disassembled. In addition, we design a variety of inspection and maintenance mechanisms, which can be simply assembled on the robot for automated operations. At the same time, a system software is developed for remote control and monitoring of the robot.

### 2.1. Design Considerations

Due to the special working environment, the inspection robot should have the capability of absorbing and climbing on vertical arc-shaped walls. As a result, a lot of issues have to be considered when designing the robot.

- **Adsorption stability:** a strong adsorption force is essential to keep the robot moving steadily on inclined or vertical walls without sliding.
- **Climbing ability:** a sufficient driving force can ensure the moving ability and load capacity of the inspection robot, especially when running on vertical surfaces.
- **Load capacity:** when carrying different equipment, the robot should overcome gravity and high-altitude resistance. Sufficient driving force and a lighter robot body can increase its load capacity.
- **Obstacle-crossing capability:** the robot should be able to smoothly pass small obstacles on the walls, e.g., weld seams, solder joints, etc.
- **Inspection and maintenance capabilities:** in order to perform automated tasks, the robot should be able to carry different equipment for inspection and maintenance.

### 2.2. Mechanical Design

**Figure 1:**
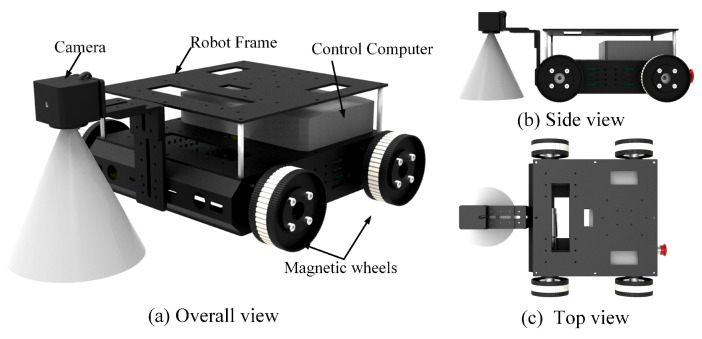
Inspection robot design.

In order to meet the design requirements, we present a robot design scheme of four bilateral-driven magnetic wheels. The robot had a lightweight body, with two motors providing sufficient driving force for moving on the walls. The prototype of the robot is shown in Figure 1 and the parameters of the robot are shown in Table 1. The dimension of the robot was 365 mm × 300 mm × 140 mm. The self-weight of the robot was 7.5 kg and the adsorption force of each magnetic wheel was 150 N. Its maximum payload and maximum climbing speed were 10.1 kg and 0.3 m/s, respectively.

**Figure 2:**
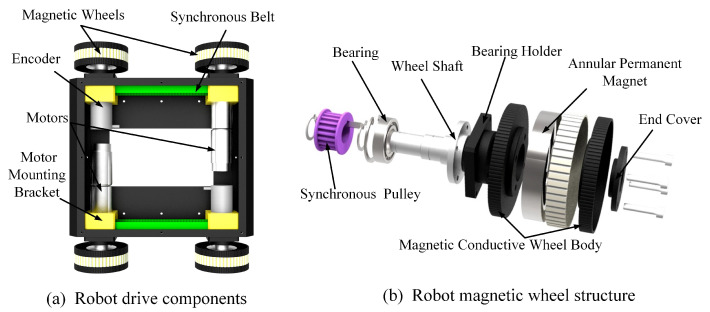
Drive component and magnetic wheel.

The designed robot was composed of four magnetic wheels, two drive components, a vision component, a multilayer robot frame and two control components. Figure 2 describes the mechanical structure of the drive component and the magnetic wheel. The drive component included a DC motor, a synchronous belt and a motor mounting bracket. The motor was installed on the robot frame through the motor mounting bracket and connected with the wheel shaft. The synchronous belt was used to drive two magnetic wheels on the same side by one motor. The design of four bilateral-driven magnetic wheels could effectively reduce the robot’s weight and volume. The encoders on both sides were used to record the robot’s climbing speed and running distance.

The modular magnetic wheel component was designed to provide sufficient adsorption force for the robot. As shown in Figure 2, the magnetic wheel was composed of a synchronous pulley, a wheel shaft, a bearing holder, a bearing, two magnetic conductive wheel bodies, a protective shell, an annular permanent magnet and an end cover. The end covers could be easily disassembled to quickly replace the magnetic wheels. The annular NdFeB permanent magnet was embedded in the wheel as the source of the adsorption force. The magnetic wheel body was designed to be as light as possible, without losing its magnetic conductive performance. The drive shaft was connected to a motor or an encoder, and the driving force could be transmitted to the magnetic wheel on the same side through the synchronous belt. The bearing holder and bearing were designed to enhance the rotation stability of the wheels and could be easily installed on the robot frame.

The vision component was used to identify weld seams and monitor the tank surface, which included a camera and two brackets. The multilayered robot frame had more space for the lithium battery and the control computer.

**Figure 3:**
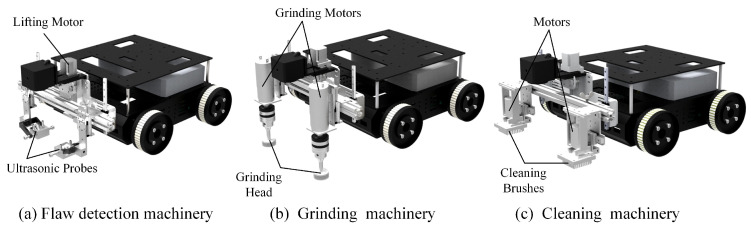
Additional machineries design.

For automated inspection and maintenance, we also investigated some additional machineries, for instance, a flaw detection machinery, grinding machinery and cleaning machinery. As shown in Figure 3a, the flaw detection machinery mainly included a lifting motor and two ultrasonic probes. The ultrasonic signals were used to detect internal defects in weld seams. The lifting motor could change the height of the ultrasonic probes. In the grinding machinery (Figure 3b) and cleaning machinery (Figure 3c), grinding heads and cleaning brushes were driven by motors. These additional machineries could be easily installed on the robot. Additional inspection and maintenance machineries effectively reduce the manual work intensity and save operating time.

## 3. Three-Dimensional Positioning of the Wall-Climbing Robot

In order to achieve the accurate spatial positioning of the wall-climbing robot, multiple-sensor information was measured and fused. The sensors used for robot positioning included an ultrawideband (UWB) sensor, an inertial measurement unit (IMU) and encoders. The UWB positioning system adopted the time-of-arrival (TOA) positioning technique to measure the absolute spatial coordinates of the robot. The IMU and the encoders were used to record the running speed and inclination angle of the robot, which could estimate the relative spatial coordinates of the robot.

### 3.1. Spatial Coordinate Measurement by UWB

**Figure 4:**
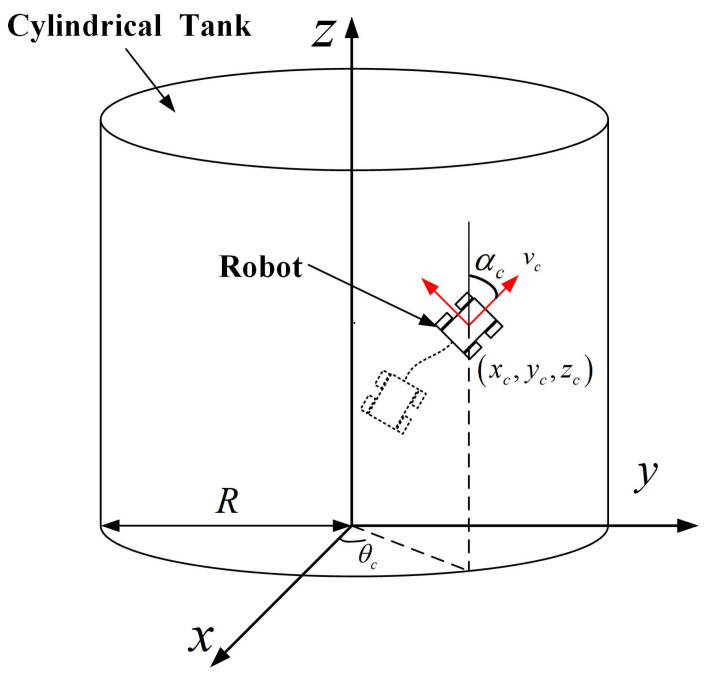
Spatial coordinate system of the robot.

The UWB positioning system included UWB robot tags and UWB anchors, which were used to estimate the spatial coordinates of the robot on the tank surface. Generally, UWB anchors are placed at certain locations with known coordinates, and the UWB robot tag is mounted on the robot. When the robot is running on the cylindrical tank, the spatial position, velocity and inclination angle of the robot are constantly changing. As shown in Figure 4, the three-dimensional Cartesian coordinate system XYZ was constructed, and the current position of the robot on the surface of the tank was $\left(x_{c},y_{c},z_{c}\right)$. *R* was the radius of the tank, and θ was the angle of the robot position on the XY coordinate plane relative to the X axis. The forward linear velocity of the robot was $v_{c}$, and the inclination angle of the robot was $\alpha_{c}$.

**Figure 5:**
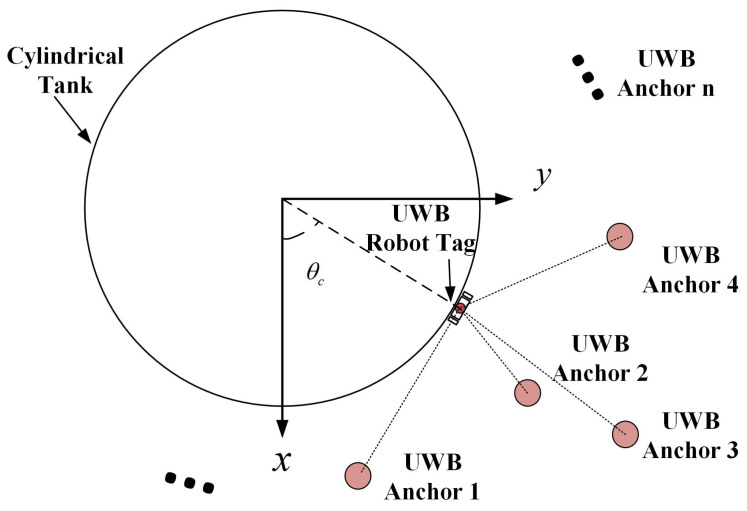
UWB positioning and coordinate calculation.

As shown in Figure 5, there were multiple UWB anchors with known coordinates $\left(x_{i},y_{i},z_{i}\right)$ around the tank, which were labelled as: Anchor 1, Anchor 2, Anchor 3, *…*, Anchor *n*, and the coordinate of the UWB robot tag was $\left(x_{c},y_{c},z_{c}\right)$. The distances between the UWB robot tag and the UWB anchors measured by the positioning system were $d_{i}\left(i=1,2,\ldots ,n\right)$. The spatial position of the robot could be obtained by four sets of valid distance values. According to the geometric relationship of the positions, the relationship between the distance $d_{i}$ and the spatial coordinate $\left(x_{i},y_{i},z_{i}\right)$ was expressed as:

(1) $$\left\{\begin{array}{c} {\left(x_{1}-x_{c}\right)}^{2}+{\left(y_{1}-y_{c}\right)}^{2}+{\left(z_{1}-z_{c}\right)}^{2}=d_{1}^{2}\\ {\left(x_{2}-x_{c}\right)}^{2}+{\left(y_{2}-y_{c}\right)}^{2}+{\left(z_{2}-z_{c}\right)}^{2}=d_{2}^{2}\\ {\left(x_{3}-x_{c}\right)}^{2}+{\left(y_{3}-y_{c}\right)}^{2}+{\left(z_{3}-z_{c}\right)}^{2}=d_{3}^{2}\\ {\left(x_{4}-x_{c}\right)}^{2}+{\left(y_{4}-y_{c}\right)}^{2}+{\left(z_{4}-z_{c}\right)}^{2}=d_{4}^{2} \end{array}\right.$$

By calculating the difference between the equations, we had:

(2) $$\left\{\begin{array}{c} 2x_{c}\left(x_{2}-x_{1}\right)+2y_{c}\left(y_{2}-y_{1}\right)+2z_{c}\left(z_{2}-z_{1}\right)=d_{1}^{2}-d_{2}^{2}+x_{2}^{2}-x_{1}^{2}+y_{2}^{2}-y_{1}^{2}+z_{2}^{2}-z_{1}^{2}\\ 2x_{c}\left(x_{3}-x_{1}\right)+2y_{c}\left(y_{3}-y_{1}\right)+2z_{c}\left(z_{3}-z_{1}\right)=d_{1}^{2}-d_{3}^{2}+x_{3}^{2}-x_{1}^{2}+y_{3}^{2}-y_{1}^{2}+z_{3}^{2}-z_{1}^{2}\\ 2x_{c}\left(x_{4}-x_{1}\right)+2y_{c}\left(y_{4}-y_{1}\right)+2z_{c}\left(z_{4}-z_{1}\right)=d_{1}^{2}-d_{4}^{2}+x_{4}^{2}-x_{1}^{2}+y_{4}^{2}-y_{1}^{2}+z_{4}^{2}-z_{1}^{2} \end{array}\right.$$

which could be described in the following matrix form:

(3) $$A_{uwb}C_{uwb}=D_{uwb}$$

where

(4) $$\begin{array}{cc} \; & A_{uwb}=\left[\begin{array}{ccc} 2\left(x_{2}-x_{1}\right) & 2\left(y_{2}-y_{1}\right) & 2\left(z_{2}-z_{1}\right)\\ 2\left(x_{3}-x_{1}\right) & 2\left(y_{3}-y_{1}\right) & 2\left(z_{3}-z_{1}\right)\\ 2\left(x_{4}-x_{1}\right) & 2\left(y_{4}-y_{1}\right) & 2\left(z_{4}-z_{1}\right) \end{array}\right]\\ \; & C_{uwb}={\left[\begin{array}{ccc} x_{c} & y_{c} & z_{c} \end{array}\right]}^{T}\\ \; & D_{uwb}=\left[\begin{array}{c} d_{1}^{2}-d_{2}^{2}+x_{2}^{2}-x_{1}^{2}+y_{2}^{2}-y_{1}^{2}+z_{2}^{2}-z_{1}^{2}\\ d_{1}^{2}-d_{3}^{2}+x_{3}^{2}-x_{1}^{2}+y_{3}^{2}-y_{1}^{2}+z_{3}^{2}-z_{1}^{2}\\ d_{1}^{2}-d_{4}^{2}+x_{4}^{2}-x_{1}^{2}+y_{4}^{2}-y_{1}^{2}+z_{4}^{2}-z_{1}^{2} \end{array}\right] \end{array}$$

Then, the coordinates of the UWB robot tag was:

(5) $$C_{uwb}={\left(A_{uwb}{}^{T}A_{uwb}\right)}^{-1}A_{uwb}{}^{T}D_{uwb}$$

**Figure 6:**
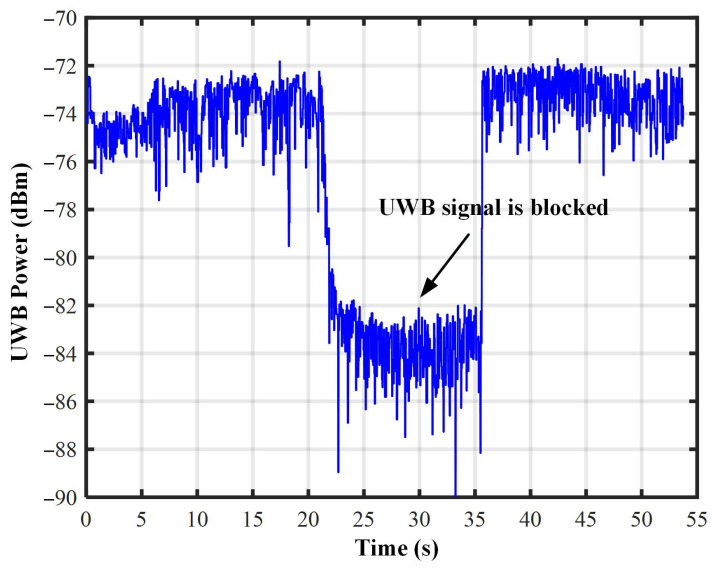
First path power of UWB signals under occlusion.

Therefore, the robot coordinate $\left(x_{c},y_{c},z_{c}\right)$ could be estimated based on the measurement data of four UWB anchors. Due to the installation positions of UWB anchors about the cylindrical tank, the signal of the UWB anchors could be blocked, and some errors could occur. As shown in Figure 6, when the UWB signal was blocked, the power of the first path decreased from about −74 dBm to −84 dBm. By analysing the power level of the first path (signal receiving power of the direct path), the availability of UWB anchor measurement data could be determined. The best four sets of measurement data were selected to calculate the spatial coordinates of the robot. The power level of the first path directly reflected the state of the propagation path of the first path signal received by the UWB anchor. When the robot ran out of the range of a UWB anchor, the power of the first path was significantly reduced. As a result, the measurement data could be inaccurate, and this UWB anchor was considered to be invalid. If there were less than four available UWB anchors to be used by the robot, the UWB positioning system became invalid.

In order to facilitate the calculation and estimation of the robot coordinates, the position of the robot in the spatial Cartesian coordinate system (x,y,z) was transformed into the coordinates of the cylindrical coordinate system (R,θ,h), where *R* is the radius of the cylinder, which is a constant, θ is the angle of the robot position relative to the X coordinate axis, and *h* is the height of the robot on the Z coordinate axis. The conversion relationship from (x,y,z) to (θ,h) is described in Algorithm 1. In order to reduce the influence of disturbance, when |x|<0.03, the robot was considered to be on the Y coordinate axis. By means of the UWB positioning technique, the cylindrical coordinates of the robot were calculated.
**Algorithm 1** Solve robot cylindrical coordinates.**Input:** $x_{c},y_{c},z_{c}$**Output:** $\theta_{c},h_{c}$1:**for** robot coordinates **do**2:   $h_{c}=z_{c}$;3:   **if** $x_{c}<0.03$ **then**4:     **if** $y_{c}\geq 0$ **then**5:        $\theta_{c}=\pi /2$;6:     **else**7:        $\theta_{c}=-\pi /2$;8:     **end if**9:   **else**10:     $\vartheta =arctan|y_{c}/x_{c}|$;11:     **if** $x_{c}>0$ and $y_{c}\geq 0$ **then**12:        $\theta_{c}=\vartheta$;13:     **else if** $x_{c}<0$ and $y_{c}\geq 0$ **then**14:        $\theta_{c}=\pi -\vartheta$;15:     **else if** $x_{c}<0$ and $y_{c}<0$ **then**16:        $\theta_{c}=-\pi +\vartheta$;17:     **else if** $x_{c}>0$ and $y_{c}<0$ **then**18:        $\theta_{c}=-\vartheta$;19:     **end if**20:   **end if**21:**end for**

### 3.2. Multisensor Positioning Data Fusion

Since different kinds of sensors have their own errors and defects, multisensor data fusion methods were used to reduce errors and obtain a higher positioning accuracy. By complementing the data from different sensors, a higher precision spatial positioning of the wall-climbing robot could be obtained. Specifically, the cumulative error generated by the encoders and IMU could be corrected by the UWB positioning data, and the encoders and IMU positioning data could also compensate for the low accuracy of the UWB positioning.

During the robot positioning system, the cylindrical absolute coordinate $\left(\theta_{c},h_{c}\right)$ could be computed by the UWB positioning subsystem. At the same time, the rotational speeds of the wheels were recorded by the encoders. Based on robot kinematics, the forward linear velocity $v_{c}$ and rotational angular velocity $\omega_{c}$ of the robot was expressed as:

(6) $$\begin{array}{cc} \; & v_{c}=\frac{r}{2}\cdot \phi_{1}+\frac{r}{2}\cdot \phi_{2}\\ \; & \omega_{c}=\frac{r}{l}\cdot \phi_{1}-\frac{r}{l}\cdot \phi_{2} \end{array}$$

where $\phi_{i}\left(i=1,2\right)$ is the angular velocity of the robot’s wheels, *r* is the radius of the magnetic wheels, and *l* is the distance between the two wheels. The speeds of the magnetic wheels on the same side of the robot were equal to each other. The IMU sensor was installed on the side of the robot, and it consisted of a three-axis accelerometer, a three-axis gyroscope, and a three-axis magnetometer. According to the Madgwick algorithm [36], the inclination angle $\alpha_{c}$ of the robot on the vertical wall was measured by the IMU. When a robot moves on the surface of a cylindrical tank, the forward linear velocity $v_{c}$ and the inclination angle $\alpha_{c}$ can be used to obtain the robot’s velocities $\left({\dot{\theta }}_{c},{\dot{h}}_{c}\right)$ in the cylindrical coordinate system:

(7) $$\left\{\begin{array}{c} {\dot{\theta }}_{c}=v_{c}\cdot \sin\alpha_{c}/R\\ {\dot{h}}_{c}=v_{c}\cdot \cos\alpha_{c} \end{array}\right.$$

In order to achieve a more precise spatial positioning of the robot, the Kalman filter was used to fuse the information acquired by different sensors. As the spatial coordinate information and velocity information of the robot were obtained by the UWB sensor, encoders and IMU, the state vector at time *k* in the Kalman filter could be written as:

(8) $$X_{k}={\left[\begin{array}{cccc} \theta_{k} & {\dot{\theta }}_{k} & h_{k} & {\dot{h}}_{k} \end{array}\right]}^{T}$$

where $\left(\theta_{k},h_{k}\right)$ and $\left({\dot{\theta }}_{k},{\dot{h}}_{k}\right)$ are the coordinate components and the velocity components of the robot motion state at time *k*, respectively.

The state transition equation of the robot system was:

(9) $$X_{k}=F\cdot X_{k-1}+\Gamma \cdot w_{k-1}$$

where $X_{k-1}$ is the state vector at time k−1, F is the state transition matrix, and $\Gamma \cdot w_{k-1}$ is the noise of the robot system.

Assuming that the robot climbs on the surface of a cylindrical tank at a constant speed, then we have:

(10) $$F=\left[\begin{array}{cccc} 1 & T & 0 & 0\\ 0 & 1 & 0 & 0\\ 0 & 0 & 1 & T\\ 0 & 0 & 0 & 1 \end{array}\right]$$

(11) $$\Gamma =\left[\begin{array}{cc} T & 0\\ 1 & 0\\ 0 & T\\ 0 & 1 \end{array}\right]$$

(12) $$w_{k}=\left[\begin{array}{c} u_{x}\\ u_{y} \end{array}\right]$$

where *T* is the data acquisition cycle of different sensors, including the coordinate data $\left(\theta_{k},h_{k}\right)$ acquisition cycle $T_{1}$ and the velocity data $\left({\dot{\theta }}_{k},{\dot{h}}_{k}\right)$ acquisition sampling cycle $T_{2}$. $u_{x}$ and $u_{y}$ are Gaussian white noises with mean value zero and variance $\sigma^{2}$.

The observation equation of the robot system was:

(13) $$Z_{k}=H\cdot X_{k}+v_{k}$$

where H is the observation matrix of the system, and $v_{k}$ is the measurement noise.

Since the position information measured by the UWB sensor was $\left(\theta_{k},h_{k}\right)$, while the velocity information measured by the encoders and the IMU was $\left({\dot{\theta }}_{k},{\dot{h}}_{k}\right)$, their corresponding observation matrices were different, which could be expressed as:

(14) $$\begin{array}{cc} H_{uwb} & =\left[\begin{array}{cccc} 1 & 0 & 1 & 0\\ 1 & 0 & 1 & 0 \end{array}\right]\\ H_{encoder} & =\left[\begin{array}{cccc} 0 & 1 & 0 & 1\\ 0 & 1 & 0 & 1 \end{array}\right] \end{array}$$

Once the measurement data from any sensor were received, the Kalman filter was updated. The predictions and updates were performed in sequence, so as to obtain a better result.

Based on the state equation and measurement equation of the robot positioning system, the Kalman filter prediction equation was constructed as follows:

(15) $$X_{\left(k|k-1\right)}=F\cdot X_{\left(k-1|k-1\right)}+w_{\left(k\right)}$$

where $X_{\left(k|k-1\right)}$ is the prediction vector at time *k* predicted by the positioning data at time k−1, $X_{\left(k-1|k-1\right)}$ is the optimal estimation vector at time k−1, and $w_{\left(k\right)}$ is noise.

The covariance of the robot system at time *k* was

(16) $$P_{\left(k|k-1\right)}=F\cdot P_{\left(k-1|k-1\right)}\cdot \Phi^{T}+Q$$

where $P_{\left(k-1|k-1\right)}$ is the covariance matrix of the robot system at time k−1, and Q is the covariance of the system noise.

The optimal estimation vector of the robot system at time *k* was:

(17) $$\begin{array}{cc} \; & X_{\left(k|k\right)}=X_{\left(k|k-1\right)}+Kg_{\left(k\right)}\left(Z_{\left(k\right)}-H\cdot X_{\left(k\right)}\right)\\ \; & Kg_{\left(k\right)}=P_{\left(k|k-1\right)}H^{T}{\left(H\cdot P_{\left(k|k-1\right)}H^{T}+R\right)}^{-1} \end{array}$$

where $Z_{\left(k\right)}$ is the measured value; it is composed of two sets of data: $\left(\theta_{c},h_{c}\right)$ and $\left({\dot{\theta }}_{c},{\dot{h}}_{c}\right)$. The H’s corresponding to different measurement data are $H_{uwb}$ and $H_{encoder}$. $Kg_{\left(k\right)}$ is the Kalman filter gain. R is the covariance matrix of the observed noise, including $R_{uwb}$ and $R_{encoder}$. A larger R means that the observed data are more volatile and less credible, so the influence of the observed amount on the final estimation result is smaller when the state is updated; on the contrary, if R is smaller, it means the observed data are more credible and will have more influence on the result.

Under this data fusion framework, if the UWB positioning signal is temporarily lost, the state of the Kalman filter can also be updated by the encoders and the IMU sensor. The reason why these two sensors are used at the same time is because the wheels of the wall-climbing robot may be slipping. If the encoders or IMU signal is lost, UWB can be applied for positioning alone. Under normal circumstances, all sensors are functional, and they can correct and complement each other, thus improving the overall positioning accuracy.

### 3.3. Robot-Positioning Accuracy Analysis

**Figure 7:**
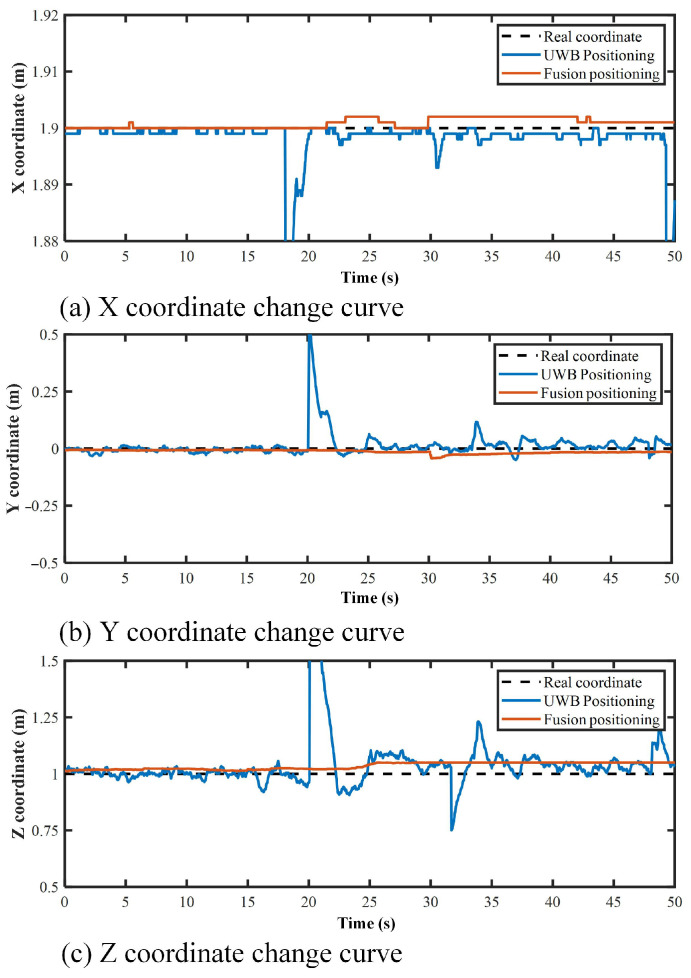
Robot’s spatial coordinates in the positioning stability experiments.

To verify the accuracy and stability of the fusion positioning method, positioning stability experiments were implemented. In the experiments, the robot rested at the spatial Cartesian coordinate point (1.9, 0, 1). Figure 7 shows the comparison of the robot coordinate stability using two positioning methods, UWB positioning (positioning only by the UWB subsystem) and fusion positioning (positioning by the UWB subsystem, IMU, encoders), respectively. Obviously, the UWB positioning error was larger, and large error peaks occurred occasionally. Since the robot was in a static state, the real coordinates of the robot were fixed. The coordinates estimated by the fusion positioning method fluctuated slightly, but the fluctuations generated by the UWB sensor were basically eliminated, and the robot positioning stability was enhanced.

**Figure 8:**
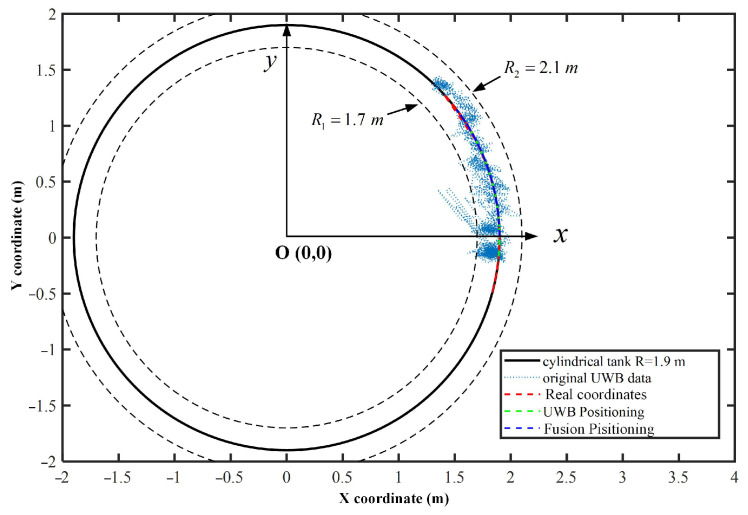
Robot’s XY coordinates in the horizontal positioning experiments.

Furthermore, motion-positioning experiments were performed to test the positioning accuracy of the robot when it moved along the arc surface of the cylindrical tank. In the experiment, the robot started from the initial spatial Cartesian coordinate point (1.9, 0, 1), moved laterally along the storage tank and returned to the initial point. The trajectory of the robot was an arc in space. Within the positioning range of the UWB positioning system, the two positioning results were tested and compared, respectively. Figure 8 illustrates the XY coordinates of the two positioning results. A circle with a radius of 1.9 m represented a cylindrical surface. The original UWB positioning results were distributed on both sides of the tank, and the error of the initial UWB positioning data in the direction of the cylinder radius was ±0.2 m. After data filtering and data conversion, the results of fusion positioning basically stayed on the circle with a radius of 1.9 m, which meant that for a moving wall-climbing robot, the fusion positioning method was able to guarantee the accuracy and stability of the positioning.

## 4. Backstepping Sliding-Mode Control

### 4.1. Kinematic Analysis

**Figure 9:**
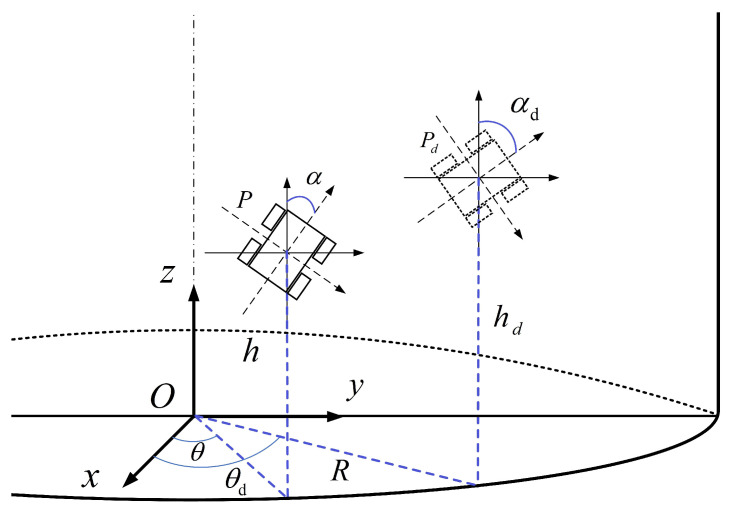
Tracking analysis of the robot.

As shown in Figure 9, in a cylindrical coordinate system with origin *O*, polar axis *x* and longitudinal axis *z*, we assumed a centre of mass of the mobile robot located at point P(R,θ,h), where *R* is the radius of the base circle of the cylinder, θ is the angular coordinate and *h* is the height.

Based on differential-drive wheels, the robot moved under nonholonomic constraints. Assembled encoders were used to measure the angular velocities of two driving wheels. Two independent PID controllers were developed to accurately control the speeds. During the robot motion process, the simplified expression of the robot kinematics was given as below:

(18) $$\begin{array}{cc} \dot{h} & =v\cdot \cos\alpha \\ \dot{\theta } & =\frac{1}{R}\cdot v\cdot \sin\alpha \\ \dot{\alpha } & =\omega \end{array}$$

where α is the robot’s orientation angle, *v* is the linear velocity and *w* is the angular velocity. The vector $q={\left[h\;\theta \;\alpha \right]}^{T}$ describes the pose (i.e., position and orientation) of the mobile robot on a cylindrical surface.

In order to make sure that the desired trajectory satisfied the no-slipping, pure-rolling condition, it was created by a virtual reference robot with pose $q_{d}={\left[h_{d}\;\theta_{d}\;\alpha_{d}\right]}^{T}$ and the desired linear velocities $v_{d}\neq 0$; the reference robot was given by

(19) $$\begin{array}{cc} {\dot{h}}_{d} & =v_{d}\cdot \cos\alpha_{d}\\ {\dot{\theta }}_{d} & =\frac{1}{R}\cdot v_{d}\cdot \sin\alpha_{d}\\ {\dot{\alpha }}_{d} & =\omega_{d} \end{array}$$

where $v_{d}$ and $w_{d}$ are the desired linear velocity and angular velocity, respectively.

### 4.2. Tracking Error Analysis

The tracking errors in the trajectory tracking problem can be described as $q_{e}={\left[h_{e}\;\theta_{e}\;\alpha_{e}\right]}^{T}$. The error matrix or mismatch matrix can be written as

(20) $$\begin{array}{c} \left[\begin{array}{c} h_{e}\\ \theta_{e}\\ \alpha_{e} \end{array}\right]=\left[\begin{array}{ccc} \cos\alpha & R\sin\alpha & 0\\ -\frac{1}{R}\sin\alpha & \cos\alpha & 0\\ 0 & 0 & 1 \end{array}\right]\left[\begin{array}{c} h_{d}-h\\ \theta_{d}-\theta \\ \alpha_{d}-\alpha \end{array}\right] \end{array}$$

Taking the time derivative of Equation (20) and substituting in Equations (18) and (19), the corresponding error derivatives are obtained:

(21) $$\begin{array}{cc} {\dot{h}}_{e} & =r\cdot \theta_{e}\cdot w-v+v_{d}\cdot \cos\alpha_{e} \end{array}$$

(22) $$\begin{array}{cc} {\dot{\theta }}_{e} & =\frac{1}{R}\left(-h_{e}\cdot w+v_{d}\cdot \sin\alpha_{e}\right) \end{array}$$

(23) $$\begin{array}{cc} {\dot{\alpha }}_{e} & =w_{d}-w \end{array}$$

### 4.3. Backstepping Sliding-Mode Control

**Lemma** **1** ([37]). *For any x∈R and |x|<∞,φ(x)=x·sin(arctanx)≥0, and the equality occurs only if x=0.*

This conclusion was used to design the switching function of the controller in the following part.

The object of the designed SMC controller was to decrease the error ${\left[h_{e}\;\theta_{e}\;\alpha_{e}\right]}^{T}$ to zero. The backstepping method was chosen to design our controller. When the height error $h_{e}=0$, define the Lyapunov function:

(24) $$V_{\theta }=\frac{1}{2}{\theta_{e}}^{2}$$

Taking the derivative of Equation (24), we have

(25) $$\dot{V_{\theta }}=\theta_{e}\cdot {\dot{\theta }}_{e}=\theta_{e}\cdot \frac{1}{R}\left(-h_{e}\cdot w+v_{d}\cdot \sin\alpha_{e}\right)$$

The system is asymptotically stable if $\dot{V_{\theta }}\leq 0$. Assume $\alpha_{e}=-\arctan\left(\frac{1}{R}\cdot v_{d}\cdot \theta_{e}\right)$, Equation (25) can be written as:

(26) $$\begin{array}{cc} \dot{V_{\theta }} & =\frac{1}{R}\left(-h_{e}\cdot \theta_{e}\cdot w+v_{d}\cdot \theta_{e}\cdot \sin\left(-\arctan\left(\frac{1}{R}\cdot v_{d}\cdot \theta_{e}\right)\right)\right)\\ \; & =\frac{1}{R}\left(-h_{e}\cdot \theta_{e}\cdot w-v_{d}\cdot \theta_{e}\cdot \sin\left(\arctan\left(\frac{1}{R}\cdot v_{d}\cdot \theta_{e}\right)\right)\right) \end{array}$$

If the system enters the sliding mode, to guarantee $h_{e}=0$ and $\alpha_{e}=-\arctan\left(\frac{1}{R}\cdot v_{d}\cdot \theta_{e}\right)$, a switching function is presented as follows:

(27) $$s=\left[\begin{array}{c} s_{1}\\ s_{2} \end{array}\right]=\left[\begin{array}{c} \dot{h_{e}}+C_{1}\cdot h_{e}\\ \alpha_{e}+\arctan\left(\frac{1}{R}\cdot v_{d}\cdot \theta_{e}\right) \end{array}\right]$$

where $C_{1}$ is a real positive parameter. The stability of the sliding surfaces $s_{1}=0$ and $s_{2}=0$ are proved as follows:

- If $s_{1}$ converges to zero, $h_{e}$ decreases to zero exponentially with a time constant determined by $C_{1}$;
- If $s_{2}$ converges zero, $\alpha_{e}\to -\arctan\left(\frac{1}{R}\cdot v_{d}\cdot \theta_{e}\right)$. Based on Lyapunov function $V_{\theta }$, error differential equation Equation (22) and Lemma 1, we can obtain that when $h_{e}\to 0$ and $\alpha_{e}\to -\arctan\left(\frac{1}{R}\cdot v_{d}\cdot \theta_{e}\right)$, $\theta_{e}$ converges to zero, which means $\alpha_{e}$ converges to zero as well.

The system states goes to the sliding surface s=0 within a limited time under control. The tracking error $q_{e}={\left[h_{e}\;\theta_{e}\;\alpha_{e}\right]}^{T}$ converges to zero as t→∞, due to the asymptotic stability of this sliding mode. If the system states tend to stay on the sliding surface, the condition $s^{T}\cdot s<0$ must be satisfied, and a generally used reaching law is described as:

(28) $$\begin{array}{cc} {\dot{s}}_{1} & =-Q_{1}\cdot s_{1}-P_{1}\cdot sgn\left(s_{1}\right) \end{array}$$

(29) $$\begin{array}{cc} {\dot{s}}_{2} & =-Q_{2}\cdot s_{2}-P_{2}\cdot sgn\left(s_{2}\right) \end{array}$$

where *P* and *Q* are the positive integer constants.

From Equation (27), we have the derivative of $s_{1}$

(30) $${\dot{s}}_{1}={\ddot{h}}_{e}+C_{1}\cdot {\dot{h}}_{e}$$

According to Equation (21), ${\ddot{h}}_{e}$ can be obtained:

(31) $${\ddot{h}}_{e}=R\cdot {\dot{\theta }}_{e}\cdot w+R\cdot \theta_{e}\cdot \dot{w}-\dot{v}+{\dot{v}}_{d}\cdot \cos\alpha_{e}-v_{d}\cdot \sin\alpha_{e}\cdot {\dot{\alpha }}_{e}$$

Substituting Equation (30) in Equation (28), we have:

(32) $${\ddot{h}}_{e}+C_{1}\cdot {\dot{h}}_{e}=-Q_{1}\cdot s_{1}-P_{1}\cdot sgn\left(s_{1}\right)$$

Then, substituting Equation (31) in Equation (32), the forward control velocity $\dot{v}$ can be calculated as:

(33) $$\begin{array}{cc} \dot{v} & =Q_{1}\cdot s_{1}+P_{1}\cdot sgn\left(s_{1}\right)+C_{1}\cdot {\dot{h}}_{e}+{\dot{v}}_{d}\cdot \cos\alpha_{e}\\ \; & -v_{d}\cdot \sin\alpha_{e}\cdot {\dot{\alpha }}_{e}+R\cdot {\dot{\theta }}_{e}\cdot w+R\cdot \theta_{e}\cdot \dot{w} \end{array}$$

In this paper, we considered the desired forward velocity $v_{d}$ as a constant. From Equation (27), the derivative of $s_{2}$ is

(34) $${\dot{s}}_{2}=\dot{\alpha_{e}}+\frac{1}{R}\cdot \frac{v_{d}}{1+(\frac{1}{R}\cdot v_{d}\cdot \theta_{e})}\cdot {\dot{\theta }}_{e}$$

To simplify the formula, let $K=\frac{1}{R^{2}}\cdot \frac{v_{d}}{1+(\frac{1}{R}\cdot v_{d}\cdot \theta_{e})}$, substituting *K* in Equation (34), ${\dot{s}}_{2}$ can be expressed as:

(35) $${\dot{s}}_{2}=\dot{\alpha_{e}}+R\cdot K\cdot {\dot{\theta }}_{e}$$

and substituting Equation (35) in Equation (29), we can obtain the following equation:

(36) $$\dot{\alpha_{e}}+R\cdot K\cdot {\dot{\theta }}_{e}=-Q_{2}\cdot s_{2}-P_{2}\cdot sgn\left(s_{2}\right)$$

Similarly, substituting Equations (22) and (23) in Equation (36), the steering control velocity *w* is given by

(37) $$w=\frac{w_{d}+K\cdot v_{d}\cdot \sin\alpha_{e}+Q_{2}\cdot s_{2}+P_{2}\cdot sgn\left(s_{2}\right)}{1+K\cdot h_{e}}$$

Therefore, based on the chosen sliding surfaces, the control law was designed to guarantee the asymptotic position and heading direction tracking with the reference trajectory. Equations (33) and (37) are the controllers for the kinematic model.

### 4.4. Stability Analysis

Let us define *V* as a Lyapunov like function, which is given by $V=\frac{1}{2}\cdot s^{T}\cdot s$. The time derivative of the function is given as

(38) $$\begin{array}{cc} \dot{V} & =s_{1}\cdot {\dot{s}}_{1}+s_{2}\cdot {\dot{s}}_{2}\\ \; & =-s^{T}\cdot Q\cdot s-P_{1}\cdot \left|s_{1}\right|-P_{2}\cdot \left|s_{2}\right| \end{array}$$

For a stable control law, $\dot{V}\leq 0$. For $Q_{i},P_{i}\geq 0$, $\dot{V}$ is negative semidefinite.

### 4.5. Numerical Simulation

Numerical simulations were performed to verify the feasibility of the spatial trajectory tracking controller. In the simulation, the maximum forward linear velocity of the robot was 0.23 m/s, and the maximum rotational angular velocity was 0.94 rad/s. The simulation parameters were set as follows: the initial height error was 0.1 m, the angular coordinate error was −0.05 rad, and the rotation angle error was π/4 rad. The robot tracked with a forward linear velocity of 0.15 m/s. The controller parameters were as follows: $C_{1}=0.5,Q_{1}=1.5,P_{1}=0.02,Q_{2}=2$ and $P_{2}=0.01$.

**Figure 10:**
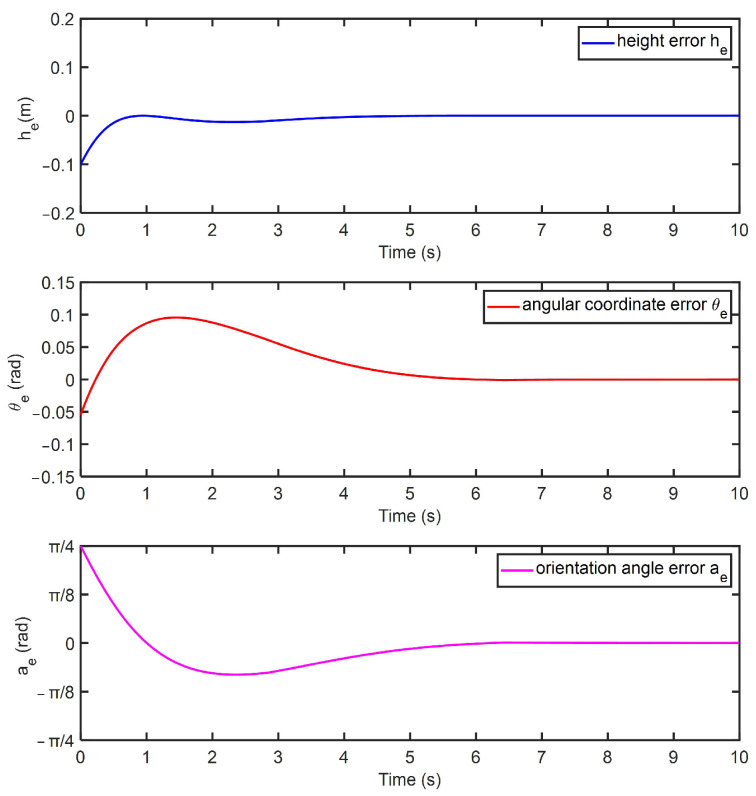
Simulation results.

Figure 10 shows the simulation results of the robot tracking the set point. The height error $h_{e}$, angular coordinate error $\theta_{e}$ and the rotation angle error $\alpha_{e}$ of the robot gradually converged to zero. The simulation results indicated that the designed controller could adjust the robot velocities according to the error and the robot could successfully track the spatial trajectory. In the subsequent experiments, the relevant parameters of the tracking controller were optimized and adjusted to ensure the actual wall-climbing robot stably tracked the spatial trajectory on tank surfaces.

## 5. Prototype Experiments

**Figure 11:**
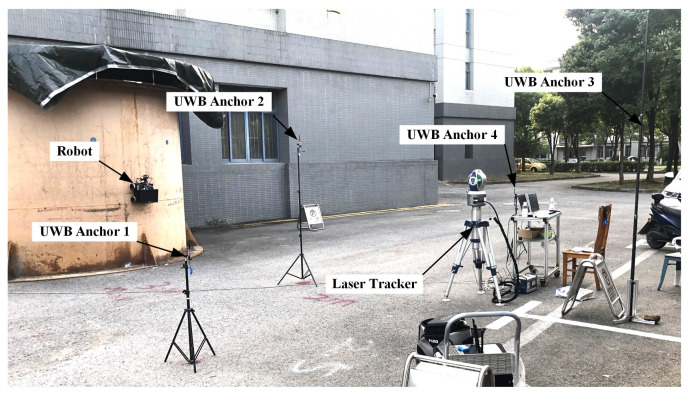
Spatial tracking experiments of the wall-climbing robot.

In order to verify the tracking control performance of the real robot on the tank surface, we conducted experiments with the wall-climbing robot on a laboratory platform. As shown in Figure 11, the experimental platform was a cylindrical tank with a diameter of 3.8 m, a height of 2.8 m and a thickness of 10 mm. The cylindrical tank was welded by multiple arc-shaped plates. UWB sensors were distributed and installed around the tank. In order to ensure the accuracy of positioning, the positions of the UWB anchors were relatively scattered, and the heights of the UWB anchors were also different. The coordinate positions of each UWB anchor in the tank coordinate system were: (6, −3, 1), (5, 0, 1.9), (9 ,0, 4) and (5, 3, 1.3). During the experiment, four UWB anchors could only cover one side of the tank, a larger number of UWB anchors could increase the coverage of the UWB positioning. A laser tracker was used to measure and record the real spatial coordinates of the robot.

The spatial trajectory tracking experiments included longitudinal trajectory tracking and horizontal trajectory tracking. In the spatial trajectory tracking experiments, the robot was adsorbed on the vertical arc-shaped wall of the cylindrical tank and its spatial position was measured according to the fusion positioning system. During the spatial trajectory tracking, the real-time position coordinates and rotation angles of the robot were recorded.

### 5.1. Longitudinal Trajectory Tracking

**Figure 12:**
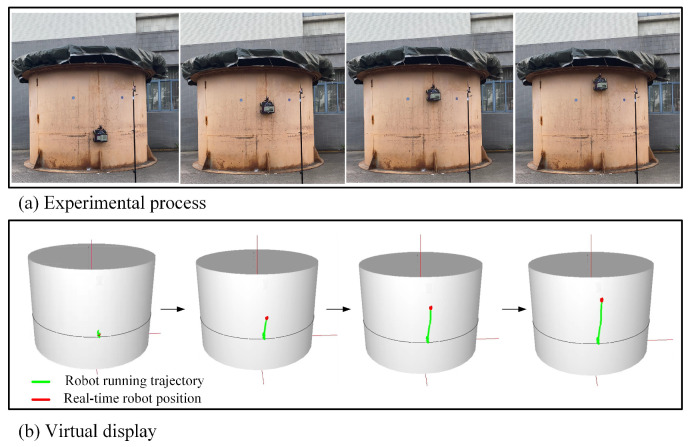
Spatial longitudinal trajectory tracking experiments.

In the spatial longitudinal trajectory tracking experiments, the starting point of the robot was selected as $\left(1.9,\;0^{\circ }\;,1\right)$ in the cylindrical coordinate system, the robot was expected to track a selected spatial longitudinal trajectory (upward along the h coordinate axis), and the desired forward linear velocity was 0.15 m/s. Figure 12 demonstrates the process of the experiments. The robot’s spatial position and running trajectory is displayed in real time on the virtual storage tank, where the red dot represents the real-time spatial position of the robot, and the green line segment borders the robot’s running trajectory.

**Figure 13:**
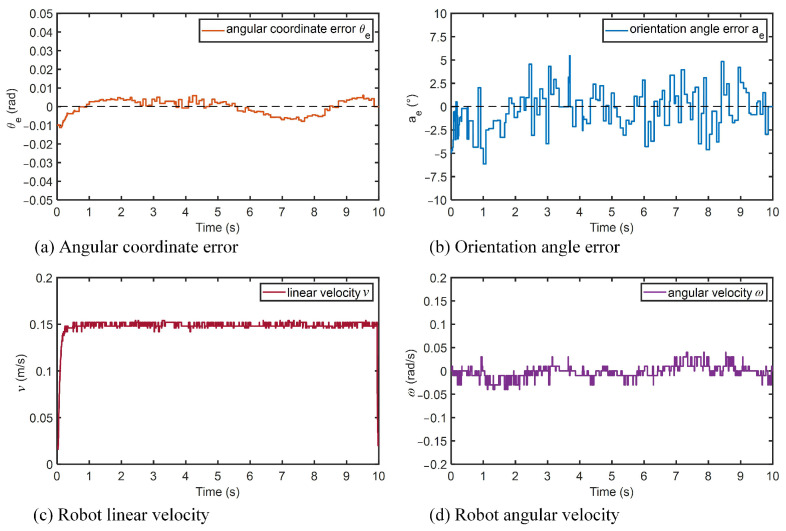
Robot tracking results of spatial longitudinal trajectory.

Figure 13 shows the results of the robot’s spatial longitudinal trajectory tracking experiments. According to the desired spatial trajectory, as the robot’s height coordinates increase, the arc angle and inclination angle of the robot should converge to the desired value of zero. Because of the limitations of the motor, there was a certain error in the speed of each motor, and the posture of the robot had slight oscillations. As shown in Figure 13a, in the tracking experiments, the robot’s angular coordinate error had a slight fluctuation, but could be quickly and continuously corrected. The maximum angular coordinate error was 0.01 rad; if converted to the three-dimensional Cartesian coordinate system XYZ, the maximum Y coordinate error of the robot was 0.019 m. In Figure 13b, we can see that the orientation angle error of the robot was also continuously adjusted during the tracking process, and it was kept within ±5${}^{\circ }$. During the tracking process, the linear velocity of the robot was basically maintained at about 0.15 m/s, with small fluctuations. The angular velocity was continuously adjusted by the controller to reduce the angular coordinate error and orientation angle error. The experimental results indicated that the designed BSMC tracking controller enabled the robot to track the spatial longitudinal trajectories with an excellent stability and precision.

### 5.2. Horizontal Trajectory Tracking

**Figure 14:**
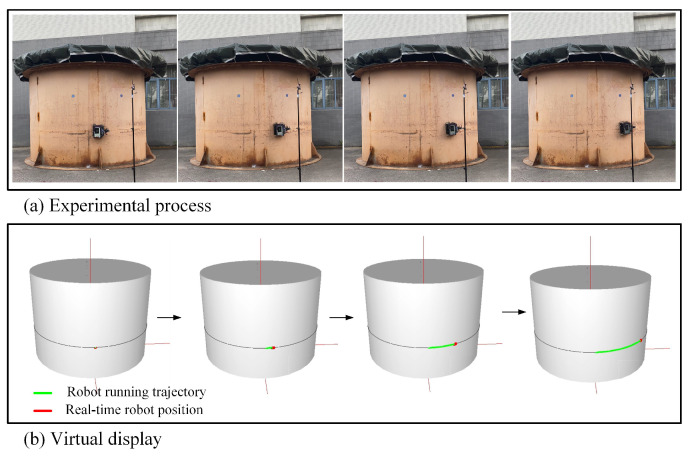
Spatial horizontal trajectory tracking experiments.

As illustrated in Figure 14, in the spatial horizontal trajectory tracking experiments, the robot tracked a horizontal arc trajectory on the tank surface. Similar to the spatial longitudinal trajectory tracking experiments, the starting point of the robot was selected as $\left(1.9,\;0^{\circ }\;,1\right)$ in the cylindrical coordinate system, the angular coordinates of the robot continued to increase when the robot was moving, and the expected height coordinates of the horizontal arc trajectory were a constant value of 1 m. The expected forward linear velocity of the robot was 0.15 m/s.

**Figure 15:**
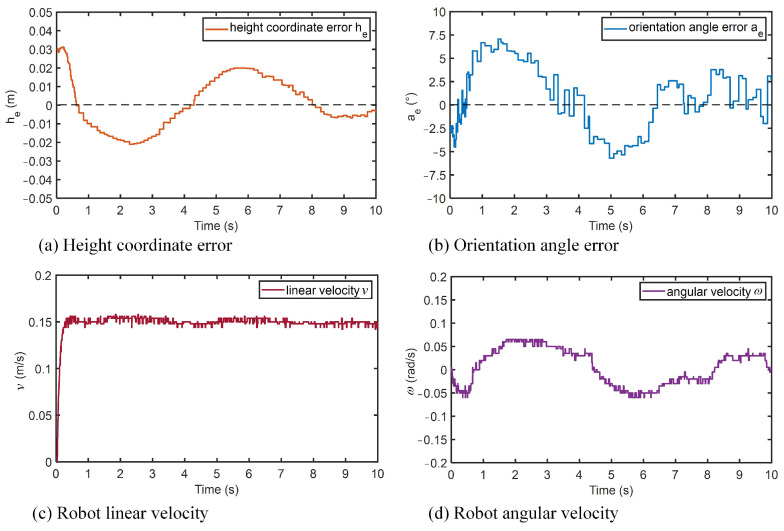
Robot’s tracking results on a spatial horizontal trajectory.

The robot height error and orientation angle error are shown in Figure 15a,b. Since the initial height error was 0.3 m, and the orientation angle error was −2.5${}^{\circ }$, the robot tracking error had large fluctuations at the beginning. Moreover, during the horizontal movement of the wall-climbing robot, due to the influence of gravity, there were slight slips that led to errors. Through a continuous adjustment by the controller, the height error and orientation angle error of the robot were continuously reduced. In the stable tracking stage, the height coordinate error of the robot was kept within ±0.02 m, and the orientation angle error was ±6${}^{\circ }$. During the tracking process, the linear velocity of the robot basically remained stable at around 0.15 m/s (Figure 15c). Because of the large errors in the height and orientation angle of the robot, the angular velocity of the robot needed to be corrected more significantly, and there were large fluctuations in the angular velocity profile (Figure 15d). In general, the horizontal trajectory tracking could still be achieved. The experimental results show that the robot could complete the tracking of the spatial horizontal arc trajectories, at a maximum height error of 0.02 m and a maximum orientation angle error of 6${}^{\circ }$. The tracking controller based on the BSMC could realize a relatively high-precision tracking performance and could reduce the deviation due to the influence of gravity.

## 6. Conclusions

In this paper, a robotic system for cylindrical storage tank inspection was presented. The designed wall-climbing robot adopted the magnetic adsorption form to realize movement on vertical walls. The vision component and additional mechanism installed on the robot were designed for automated inspection and maintenance. In order to achieve the accurate spatial positioning of the robot, a multisensor-data-fusion positioning method was developed. The sensors used for the robot positioning included a UWB sensor, an IMU and encoders. The positioning experimental results showed that the fluctuations generated by the UWB sensor were basically eliminated, the fusion positioning method could improve the accuracy and stability of the robot positioning. In addition, a hybrid control strategy, BSMC, was proposed for spatial trajectory tracking based on the combination of backstepping control and sliding-mode control. Simulation results showed that the controller could quickly correct the errors. Meanwhile, the global asymptotic stability and performance of the control system were guaranteed. Furthermore, prototype experiments were conducted on our laboratory platform to verify the performance of the designed controller. The results indicated that the wall-climbing robot could track both longitudinal and horizontal spatial trajectories stably with a high precision. Consequently, combined with the multisensor-data-fusion positioning technique, the designed sliding-mode controller could realize a precise spatial trajectory tracking of the wall-climbing robot on the cylindrical tank.

In future work, first, we will continuously improve the comprehensive performance of the robotic system. For example, the robot’s chassis structure will be modified to accommodate surfaces with different curvatures in order to be used for the inspection of more types of storage tanks, such as spherical tanks. Furthermore, we will continue to improve the anti-interference ability and fast tracking ability of the controller, so as to complete the inspection task in a shorter time.

## Figures and Tables

**Table 1:** Parameters of the wall-climbing robot.

Parameter	Value
Dimension	365×300×140mm
Wheel diameter	89mm
Mass	7.5kg
Payload	10.1kg
Maximum climbing speed	0.3m/s
Motor power	120W
Adsorption force	600N

## Data Availability

All data included in this study are available upon request by contact with the corresponding author.

## References

[1] Briones L., Bustamante P., Serna M.A. Wall-climbing robot for inspection in nuclear power plants. Proceedings of the 1994 IEEE International Conference on Robotics and Automation.

[2] Kalra L.P., Gu J., Meng M. A wall climbing robot for oil tank inspection. Proceedings of the 2006 IEEE International Conference on Robotics and Biomimetics.

[3] Song Y.K., Lee C.M., Koo I.M., Tran D.T., Moon H., Choi H.R. Development of wall climbing robotic system for inspection purpose. Proceedings of the 2008 IEEE/RSJ International Conference on Intelligent Robots and Systems.

[4] Shang J., Bridge B., Sattar T., Mondal S., Brenner A. (2008). Development of a climbing robot for inspection of long weld lines. Ind. Robot. Int. J..

[5] Fernández R., González E., Feliú V., Rodríguez A.G. A wall climbing robot for tank inspection. An autonomous prototype. Proceedings of the IECON 2010-36th Annual Conference on IEEE Industrial Electronics Society.

[6] Yu L., Yang E., Ren P., Luo C., Dobie G., Gu D., Yan X. Inspection robots in oil and gas industry: A review of current solutions and future trends. Proceedings of the 2019 25th International Conference on Automation and Computing (ICAC).

[7] Kuo C.H., Chou H.C., Chernousko F.L., Gradetsky V., Bolotnik N., Setijadi E. Trajectory tracking of a wheeled wall climbing robot using PID controller. Proceedings of the 2015 International Conference on Advanced Mechatronics, Intelligent Manufacture, and Industrial Automation (ICAMIMIA).

[8] Gimenez A., Abderrahim M., Padron V., Balaguer C. (2002). Adaptive control strategy of climbing robot for inspection applications in construction industry. IFAC Proc. Vol..

[9] Dian S., Fang H., Zhao T., Wu Q., Hu Y., Guo R., Li S. (2020). Modeling and trajectory tracking control for magnetic wheeled mobile robots based on improved dual-heuristic dynamic programming. IEEE Trans. Ind. Inform..

[10] Zhong Z., Xu M., Xiao J., Lu H. (2021). Design and control of an omnidirectional mobile wall-climbing robot. Appl. Sci..

[11] Wu X., Liu J., Zhou Y., Lv Q., Hu C. (2017). Movement control and attitude adjustment of climbing robot on flexible surfaces. IEEE Trans. Ind. Electron..

[12] Xin L., Pengli L., Yang L., Wang C. (2019). Back-Stepping Fuzzy Adaptive Sliding Mode Trajectory Tracking Control for Wall-Climbing Robot. J. Comput..

[13] Wu X., Wang C., Hua S. (2020). Adaptive extended state observer-based nonsingular terminal sliding mode control for the aircraft skin inspection robot. J. Intell. Robot. Syst..

[14] Lee G., Kim H., Seo K., Kim J., Kim H.S. (2015). MultiTrack: A multi-linked track robot with suction adhesion for climbing and transition. Robot. Auton. Syst..

[15] Sakagami N., Yumoto Y., Takebayashi T., Kawamura S. (2019). Development of dam inspection robot with negative pressure effect plate. J. Field Robot..

[16] Liu Y., Sun S., Wu X., Mei T. (2015). A wheeled wall-climbing robot with bio-inspired spine mechanisms. J. Bionic Eng..

[17] Xu F., Meng F., Jiang Q., Peng G. (2020). Grappling claws for a robot to climb rough wall surfaces: Mechanical design, grasping algorithm, and experiments. Robot. Auton. Syst..

[18] Cai Z., Tao Z., Bai J., Qu G., Zhang S. Design of landing platform on climbing robot for a Small Unmanned Aerial Vehicle. Proceedings of the 2015 IEEE International Conference on Mechatronics and Automation (ICMA).

[19] Zhou Q., Li X. (2018). Experimental investigation on climbing robot using rotation-flow adsorption unit. Robot. Auton. Syst..

[20] Faruq Howlader M.O., Sattar T.P. Novel adhesion mechanism and design parameters for concrete wall-climbing robot. Proceedings of the 2015 SAI Intelligent Systems Conference (IntelliSys).

[21] Li B., Ushiroda K., Yang L., Song Q., Xiao J. (2017). Wall-climbing robot for non-destructive evaluation using impact-echo and metric learning SVM. Int. J. Intell. Robot. Appl..

[22] La H.M., Dinh T.H., Pham N.H., Ha Q.P., Pham A.Q. (2019). Automated robotic monitoring and inspection of steel structures and bridges. Robotica.

[23] Wang R., Kawamura Y. (2016). An automated sensing system for steel bridge inspection using GMR sensor array and magnetic wheels of climbing robot. J. Sensors.

[24] Cai J., He K., Fang H., Chen H., Hu S., Zhou W. The design of permanent-magnetic wheeled wall-climbing robot. Proceedings of the 2017 IEEE International Conference on Information and Automation (ICIA).

[25] Tang C., Zhou G., Gao Z., Shu X., Chen P. (2019). A novel rail inspection robot and fault detection method for the coal mine hoisting system. IEEE Intell. Transp. Syst. Mag..

[26] Li J., Wang X.S. Novel omnidirectional climbing robot with adjustable magnetic adsorption mechanism. Proceedings of the 2016 23rd International Conference on Mechatronics and Machine Vision in Practice (M2VIP).

[27] Amakawa T., Yamaguchi T., Yamada Y., Nakamura T. Proposing an adhesion unit for a traveling-wave-type, omnidirectional wall-climbing robot in airplane body inspection applications. Proceedings of the 2017 IEEE International Conference on Mechatronics (ICM).

[28] Huang H., Li D., Xue Z., Chen X., Liu S., Leng J., Wei Y. (2017). Design and performance analysis of a tracked wall-climbing robot for ship inspection in shipbuilding. Ocean Eng..

[29] Kermorgant O. (2018). A magnetic climbing robot to perform autonomous welding in the shipbuilding industry. Robot. Comput. Integr. Manuf..

[30] Gao F., Fan J., Zhang L., Jiang J., He S. (2020). Magnetic crawler climbing detection robot basing on metal magnetic memory testing technology. Robot. Auton. Syst..

[31] Hu J., Han X., Tao Y., Feng S. (2022). A magnetic crawler wall-climbing robot with capacity of high payload on the convex surface. Robot. Auton. Syst..

[32] Teixeira M.A.S., Santos H.B., Dalmedico N., de Arruda L.V.R., de Oliveira A.S. (2018). Intelligent environment recognition and prediction for NDT inspection through autonomous climbing robot. J. Intell. Robot. Syst..

[33] Ding Y., Sun Z., Chen Q. Non-contacted permanent magnetic absorbed wall-climbing robot for ultrasonic weld inspection of spherical tank. Proceedings of the MATEC Web of Conferences. EDP Sciences.

[34] Li J., Jin S., Wang C., Xue J., Wang X. (2022). Weld line recognition and path planning with spherical tank inspection robots. J. Field Robot..

[35] Li J., Xue J., Fu D., Gui C., Wang X. (2022). Position Estimation and Error Correction of Mobile Robots Based on UWB and Multisensors. J. Sens..

[36] Madgwick S.O., Harrison A.J., Vaidyanathan R. Estimation of IMU and MARG orientation using a gradient descent algorithm. Proceedings of the 2011 IEEE International Conference on Rehabilitation Robotics.

[37] Wu W.G., Chen H.T., Wang Y.J. (2001). Global trajectory tracking control of mobile robots. Acta Autom. Sin..

